# Potential focal drivers of atrial fibrillation at the left atrial roof vein

**DOI:** 10.1016/j.hrcr.2021.10.003

**Published:** 2021-10-15

**Authors:** Hideyuki Hasebe, Yoshitaka Furuyashiki

**Affiliations:** ∗ Department of Cardiology, Faculty of Medicine, University of Tsukuba, Tsukuba, Japan; † Division of Arrhythmology, Shizuoka Saiseikai General Hospital, Shizuoka, Japan

**Keywords:** Ablation, Atrial fibrillation, CARTOFINDER, Driver, Roof vein

## Introduction

Continued development of ablation technologies has improved the efficacy of catheter ablation for paroxysmal atrial fibrillation (PAF). However, a small but significant number of patients cannot achieve freedom from atrial fibrillation (AF) owing to non–pulmonary vein (PV) triggers or drivers. Although there remains great controversy surrounding the mechanisms of AF, localized sources in the form of focal discharges or rotors may sustain AF. CARTOFINDER (Biosense Webster, Inc, Diamond Bar, CA) is a novel mapping system that can identify potential drivers of AF by mapping wavefront propagation using multipolar catheters.^1^^,^^2^

A left atrial (LA) roof vein is a rare anatomic variant of PVs, and thus, its arrhythmogenicity has not been well studied.^3^ We report a case of PAF in a patient in whom the CARTOFINDER module map revealed potential drivers with a focal repetitive activation pattern at the LA roof vein. Radiofrequency (RF) applications encircling the roof vein terminated the AF.

## Case report

**Figure 1:**
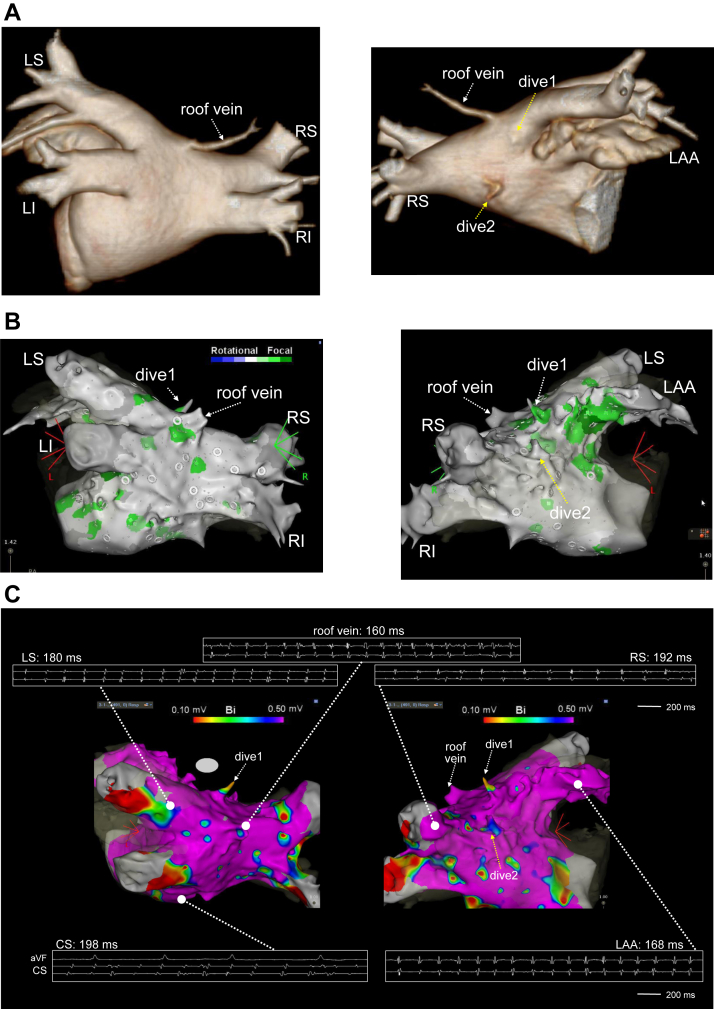
**A:** Three-dimensional computed tomography images showed a left atrial (LA) roof vein at the middle of the LA roof. In addition, there were 2 diverticula in the LA (dive1 and dive2). **B:** CARTOFINDER (Biosense Webster, Inc, Diamond Bar, CA) module map before pulmonary vein (PV) isolation. Drivers with a focal repetitive activation pattern (labeled in green) were identified in the LA appendage, PVs, septum, dive1 and the LA posterior wall, and around the roof vein. **C:** Voltage map in the LA during atrial fibrillation and cycle length at each site. The bipolar peak-to-peak voltages were >0.5 mV at the tops of dive1 and dive2. CS = coronary sinus; dive = diverticulum; LAA = left atrial appendage; LI = left inferior pulmonary vein; LS = left superior pulmonary vein; RI = right inferior pulmonary vein; RS = right superior pulmonary vein.

**Figure 2:**
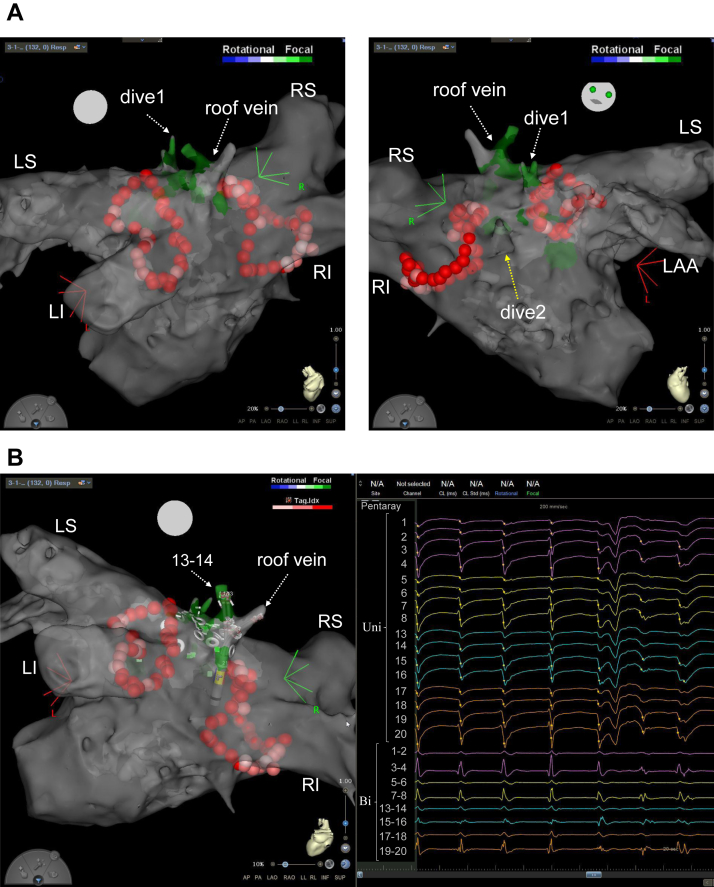
**A:** CARTOFINDER (Biosense Webster, Inc, Diamond Bar, CA) module map around the roof vein after pulmonary vein isolation. Drivers with a focal repetitive activation pattern (labeled in green) were identified inside and around the roof vein and inside dive1. **B:** Electrogram recorded on the PentaRay catheter (Biosense Webster, Inc, Diamond Bar, CA) (right panel) and catheter position at the recording (left panel). The unipolar electrograms recorded inside the roof vein (PentaRay 13 and 14) showed a QS pattern. Bi = bipolar electrocardiogram; Uni = unipolar electrocardiogram. Other abbreviations are as in Figure 1.

**Figure 3:**
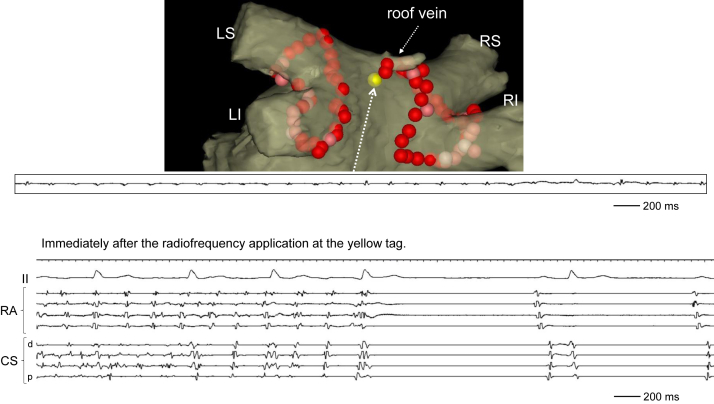
Three-dimensional image showing the ablation tags at atrial fibrillation (AF) termination (upper panel) and intracardiac electrograms immediately after the radiofrequency (RF) application at the yellow tag (lower panel). Immediately after the third RF application for encircling of the roof vein (*yellow tag*), the AF terminated. RA = right atrium. Other abbreviations are as in Figure 1.

A 60-year-old man was referred to our institution for catheter ablation for symptomatic PAF. Three-dimensional multidetector computed tomography revealed an LA roof vein at the middle of the LA roof. In addition, there were 2 diverticula in the LA: 1 (dive1) was on the left-sided roof and the other (dive2) was in the anterosuperior wall (Figure 1A). The ablation procedure was performed using a 3-dimensional navigation system (CARTO 3; Biosense Webster, Inc). The patient presented to the electrophysiology laboratory in sinus rhythm. Immediately after the transseptal puncture, burst pacing from the coronary sinus induced sustained AF. After AF had been sustained for >5 minutes, internal cardioversion was performed, but no immediate initiations of AF were observed. Again, burst pacing induced sustained AF, and the LA was mapped with CARTOFINDER using a PentaRay catheter (Biosense Webster, Inc). The PentaRay catheter was stabilized at each site for 30 seconds and 30-second unipolar and bipolar electrograms were stored in CARTO3. The CARTOFINDER module map revealed AF drivers with a focal repetitive activation pattern in the LA appendage, PVs, septum, dive1 and the LA posterior wall, and around the roof vein (Figure 1B). The bipolar peak-to-peak voltages during AF in the LA body were >0.5 mV except for those at the top of dive1 and dive2 (Figure 1C). The mean cycle length at the roof vein was the shortest in the LA (Figure 1C). Circumferential antral PV isolation (PVI) was performed with RF applications in a point-by-point fashion using a ThermoCool SmartTouch irrigated-tip contact force–sensing ablation catheter (Biosense Webster, Inc). The successful PVI did not terminate AF. The area around the roof vein was remapped with CARTOFINDER. In the remap, AF drivers with a focal repetitive activation pattern were again identified inside and around the roof vein and inside dive1 (Figure 2A and 2B). In addition to the presence of the drivers, considering that a roof vein and atrial diverticula can be potential sources of AF triggers, we decided to isolate the roof vein and dive1. First, we started isolation of the roof vein from the posterior aspect with RF energy at a power setting of 35 W and an ablation index of ≥450. Immediately after the third RF application to encircle the roof vein, the AF terminated (Figure 3A and 3B). The roof vein and dive1 were successfully isolated by the encircling RF applications. Burst pacing from the coronary sinus no longer induced sustained AF, nor did continuous isoproterenol infusion or rapid injection of adenosine triphosphate. The patient has remained asymptomatic for 6 months without any antiarrhythmic drugs. No recurrence of atrial tachyarrhythmia was detected by 24-hour Holter electrocardiogram or exercise stress electrocardiogram at 4 months after the ablation.

## Discussion

An LA roof vein, one of the rare variants of PVs, was originally reported by Igawa and colleagues.^3^ The roof vein in their report did not have histologically proven myocardial sleeves. In the present case, however, bipolar voltages inside the roof vein during AF were >0.5 mV, suggesting the existence of a myocardial sleeve similar to that of the general PVs. The drivers were reproducibly identified around the ostium of the roof vein by CARTOFINDER, indicating that these drivers were independent of the PVs. After PVI, drivers were identified not only around the roof vein but also inside it. However, direct RF applications to the drivers inside the roof vein may lead to vein perforation. In addition, the roof vein can be the source of an AF trigger.^4^^,^^5^ Therefore, we decided to isolate the roof vein by encircling it, and the RF applications around the roof vein terminated the patient’s AF. This phenomenon is consistent with the report by Honarbakhsh and colleagues,^1^ which showed that RF applications at drivers identified by CARTOFINDER both before and after PVI were associated with a higher rate of AF termination, suggesting the high contribution of these drivers to maintaining AF. It is not necessary to eliminate all focal repetitive activation patterns because some of them could be bystanders.^6^ In the present case, the RF applications around the roof vein terminated the AF and, moreover, made the AF noninducible. Therefore, we left the residual focal repetitive activation patterns alone. Although previous reports found arrhythmogenicities of the roof vein to be a trigger of AF,^4^^,^^5^ the present case suggests that the roof vein served as a perpetuator of AF. This speculation is supported by the finding that the mean cycle length at the roof vein was the shortest in the LA.

Mappings with CARTOFINDER are usually performed in combination with a PentaRay catheter. The use of PentaRay catheter might have enabled high-resolution mapping with sufficient contact in the small roof vein owing to its unique flexible spines with 1-mm multielectrodes. In addition, a roof vein would not have been detected by mapping only. Therefore, it is important to detect such anatomical variants before ablation of AF by using multidetector computed tomography or magnetic resonance imaging.

A limitation of this case report is that we analyzed AF induced by pacing, which may be different from spontaneous AF.

## Conclusion

Drivers with a focal repetitive activation pattern were identified at the LA roof vein by CARTOFINDER both before and after the PVI. Radiofrequency applications around the roof vein terminated the AF, suggesting that the roof vein served as a perpetuator of AF. Isolation of roof veins should be considered when electrical activities as triggers or perpetuators of AF are observed.

**Key Teaching Points**

• A left atrial roof vein is a rare anatomical variant of pulmonary vein (PV). Its arrhythmogenicity has not been well studied.
• CARTOFINDER is a novel mapping system that can identify potential drivers of atrial fibrillation (AF) by mapping wavefront propagation.
• Drivers with a focal repetitive activation pattern were reproducibly identified at the roof vein both before and after PV isolation, indicating that these drivers were independent of the PVs.
• Radiofrequency applications around the roof vein terminated the AF, suggesting that the roof vein served as a perpetuator of AF. Isolation of roof veins should be considered when electrical activities as triggers or perpetuators of AF are observed.
